# A purple swelling on the tongue

**DOI:** 10.11604/pamj.2015.21.234.7497

**Published:** 2015-07-31

**Authors:** Prashanth Panta

**Affiliations:** 1 Department of Oral Medicine and Radiology, MNR Dental College and Hospital, Sangareddy, Andhra Pradesh, India

**Keywords:** Benign swelling, hamartoma, vascular lesion

## Image in medicine

A painless, purple, dome shaped swelling was discovered on routine oral examination in a 20 year old male, near the right lateral border of the tongue. He revealed that the swelling had been growing in a subtle manner since 2 months, and he reported no history of trauma. It measured about 1.5x1.5 cm, surface appeared granular and was soft in consistency. Diascopic examination using a glass slide resulted in blanching, a feature characteristic of vascular and inflammatory lesions. Based on the clinical features a differential diagnosis of pyogenic granuloma, hemangioma, angiosarcoma and kaposi's sarcoma were considered. Later the lesion was excised in its entirety and histopathological examination suggested a diagnosis of ‘cavernous hemangioma’. The healing was uneventful and no recurrence was noted during a 12 month follow up period. Hemangiomas are benign hamartomatous lesions that are slow growing, sessile or pedunculated, smooth or lobulated, red swellings which sometimes exhibit a bluish hue. Hemangiomas of the tongue need special attention due to their susceptability to trauma from masticatory forces.

**Figure 1:**
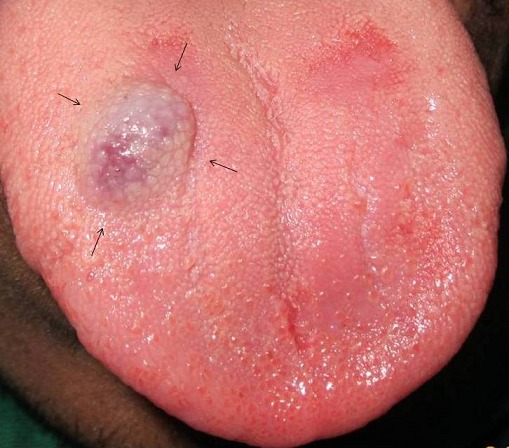
A painless purple swelling on the dorsum of the tongue

